# Long-Term Follow-Up after Prostatectomy for Prostate Cancer and the Need for Active Monitoring

**DOI:** 10.1155/2020/7196189

**Published:** 2020-03-10

**Authors:** Gregory P. Swanson, Wencong Chen, Sean Trevathan, Michael Hermans

**Affiliations:** ^1^Baylor Scott and White Health, 2401 South 31st Street Temple, Dallas, TX 76508, USA; ^2^Olin Teague VA, Temple, TX 76504, USA

## Abstract

**Background:**

Only truly long-term follow-up can determine the ultimate outcome in prostate cancer. Most studies have a median follow-up of less than 10 years and then project outcomes out to 15 and 20 years. We sought to follow patients for at least 20 years. *Materials and Methods*. We followed 754 prostate cancer patients treated with radical prostatectomy from 1988 to 1995 for a median follow-up (in survivors) of 23.9 years. We excluded lymph node and seminal vesicle positive patients and an additional 47 patients that did not have baseline prostate-specific antigen (PSA). This left 581 patients for analysis.

**Results:**

With the factors of PSA, Gleason score, and extraprostatic extension/margin positivity, we could partition patients into three risk groups for biochemical failure (low, intermediate, and high). In further analysis, we found that the risk of metastatic disease in the first two groups was almost identical (4% and 5%, respectively), while it was 19% in the high-risk group. High-risk patients were those with PSA >20 ng/ml and/or Gleason >7, or Gleason 7 + PSA 10–20 + epe (and or margin) positive. They had a 22% prostate cancer mortality.

**Conclusion:**

In patients with truly long-term follow-up after prostatectomy for prostate cancer, the risk of metastatic disease and cancer death is very low. Patients with the lower risk findings do not appear to benefit from routine follow-up after 10 years free of biochemical recurrence. With a higher risk of later failure, we recommend that the higher risk patients be followed at least intermittently for another 5 years (out to 15 years).

## 1. Introduction

Radical prostatectomy is one of the standard treatments for prostate cancer. Prostate cancer can have a long natural history; therefore, the ultimate effect of treatment can only be determined by long-term follow-up. We previously had reported on a large cohort of patients with 9.5-year median follow-up (with some patients out to 19 years) [1]. There are few studies with this length of follow-up. We have followed the same cohort for another 10 years (median 17 years) to assess the ultimate impact of prostate cancer on this group of patients and the need for long-term surveillance.

## 2. Materials and Methods

We evaluated patients undergoing radical retropubic prostatectomy between 1985 and 1995. There were a total of 754 consecutive patients operated on by a total of 6 different urological surgeons. We found the inclusion of lymph node positive patients problematic in that patients from this era who were found to have positive lymph nodes at initial exploration usually did not undergo completion prostatectomy. Those that did were more likely to have unrecognized smaller volume lymphatic disease recognized during pathologic evaluation. Also, lymphatic involvement is considered metastatic disease, with a significant risk of systemic progression and prostate cancer mortality. It is also now well recognized that seminal vesicle (SV) positive patients have an equally high risk for recurrence [2, 3]. In this current evaluation, we excluded the lymph node- and SV-positive patients from further analysis to better understand the effect of PSA, Gleason score, and other pathology factors (extraprostatic extension (epe) and positive margins). An additional 47 patients did not have a baseline PSA (mostly from the 1980's). This left 581 patients for evaluation.

The prostatectomy specimens were uniformly processed with inking of the external surface. The glands were randomly section with multiple sections from areas of known or gross disease and areas suspicious for cancer extension. Additional sections were taken from the apex and base with careful attention to the bladder neck and urethral margin. Findings were recorded as per laterality, extension into or through the capsule, seminal vesicle involvement, margin involvement, and involvement of the base or bladder neck.

Gleason staging was performed as per recommended standards and was reviewed for consistency via intradepartmental consultation. In 2005, the International Society of Urological Pathology (ISUP) revised the Gleason scoring system [4]. For this review, all the available pathology (tissue blocks and/or slides) were revised and the Gleason score was redetermined by the revised criteria by an experienced genitourinary pathologist (VOS).

There were four patients that did not have Gleason grading done, and tissue was not available for review. Patients were followed routinely every 4–6 months with a PSA and clinic visit. It was variable how long this continued in the urology clinic, with time of release back to the managing physician varying by the urologist. At that point, the obtaining of routine PSA blood tests became more variable. As other medical issues became more urgent and/or the patients' health declined, such testing often ceased.

PSA methodology changed over the years. While we cannot account for those done outside our institution, three methods have been incorporated over the last 30 years. The initial PSA assay was the Abbot (Abbot Laboratories, Abbot Park, Illinois, USA) assay until the 1996 merger with Beckman Coulter (Beckman Coulter Inc. Brea, California USA), and their hybritech assay was used until 2009 to present when we start using the newer Abbott assay. The normal range remained 0–4 ng/ml throughout. In every patient, we sought information on survival and cancer status, including PSA if available. If patients were no longer seen in our clinics, letters were sent to the local physician and/or the patients. In patients that were deceased without clear outcome information, attempts were made to obtain death certificates.

After the utility of PSA became better understood, the use of routine radiographic screening (i.e., bone scans) declined. Failure is defined as a PSA >0.3 ng/ml. The use of salvage therapy was recorded. Time to biochemical recurrence, metastatic disease, and death were recorded. Prostate cancer death was recorded as such only when the cancer contributed to death. For example, a patient that died with a rising PSA without active metastatic disease was not considered to have died from prostate cancer.

## 3. Statistical Methods

Time to overall survival was defined as time to last follow-up; time to biochemical failure is defined as time to date of first biochemical failure. If the biochemical failure is observed, it is time to the date of first biochemical failure; otherwise, it is time to date of last follow-up; time to metastasis-free survival is defined as time to date of first metastasis when the event is observed; otherwise, it is time to date of last follow-up; time to death caused by prostate cancer is defined as time to death caused of prostate cancer; otherwise, it is time to date of last follow-up. For all the four types of time to events, if we observed the event, the censor indicator is 1, otherwise it is 0.

Hazard ratios from multivariate Cox regression analysis were used to determine which risk scores are associated with biochemical-free survival. Two-sided *p* values of less than 0.05 were considered to indicate statistical significance. Assignment of points to risk factors was based on a linear transformation of the *β* regression coefficients. The coefficient of each variable was divided by 0.3, multiplied by a constant (1.88), and rounded to the nearest integer. Total point is 20. All analyses were carried out with the use of SAS software, version 9.4 (SAS Institute Inc.).

## 4. Results

A total of 754 patients underwent radical prostatectomy. There were 35 lymph node positive patients, 91 seminal vesicle positive patients, and 47 without baseline PSA who were excluded. This left 581 for evaluation (Table 1). Forty-seven (2%) did not have available tissue to reevaluate the Gleason score, so the original Gleason score was used.

Overall median follow-up is 203.6 months (17 years) and for those still alive, 286.8 months (23.9 years). A total of 458 (79%) patients are deceased. Two hundred twelve (36%) had documented PSA failure, 36 (6%) documented metastatic disease, and 44 (8%) died of prostate cancer (in 8 patients, the actual date of metastatic disease was unknown, but in the medical records and/or death certificate, prostate cancer was listed as the cause of death). Initial salvage treatment was androgen ablation and/or radiation therapy. One hundred five were known to have received radiation therapy. Sixteen received pelvic radiation therapy within the first 6 months after surgery (“adjuvant”), 13 of whom had a persistently detectable PSA. Of those still alive (*n* = 122), 88 (72%) had active follow-up in the last two years and 109 (89%) had active follow-up in the last 5 years. Overall, for all patients, 69% had a PSA within the last 5 years and 89% within the last 10 years of their most recent follow-up (dead or alive) (see supplement for follow-up details ([Supplementary-material supplementary-material-1])).

**Figure 1:**
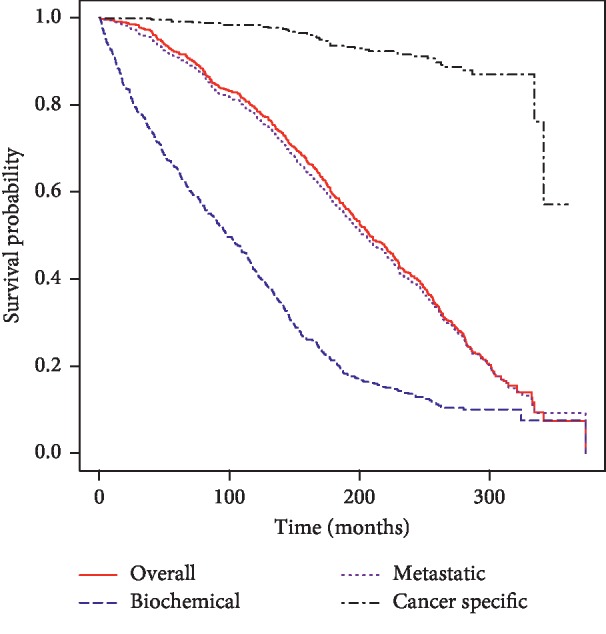
Kaplan–Meier estimate for overall survival, biochemical failure-free survival, metastatic failure-free survival, and cancer-specific survival for the entire cohort (*n* = 581).

The Kaplan–Meier estimate for overall, biochemical (PSA) failure-free, metastasis-free, and cancer-specific survival is shown in Figure 1. The 20-year Kaplan–Meier estimate for biochemical failure-free, metastasis-free, cancer-specific, and overall survival was 14%, 40%, 91%, and 41%, respectively.

Since biochemical failure is usually the initial indicator of failure, we evaluated the parameters of PSA, Gleason score, and pathologic status for risk of biochemical failure (Table 2). Instead of using the subjective clinical staging, we evaluated the more specific pathology factors. On univariate analysis, for each PSA risk group, the risk of biochemical failure increased significantly from a PSA of <10 ng/ml (28% risk) to PSA of 10–20 ng/ml (54% risk) to PSA >20 ng/ml with a 76% risk (hazard ratio 3.97). Similar increases were seen with the Gleason score: from the Gleason <7 failure rate of 28% to 38% with Gleason 7 and 73% with Gleason >7 (HR 2.85). Having either epe or a positive margin increased the risk from 29% to 60% with an HR of 1.64.

On multivariate analysis (Table 3), all of the above parameters remained significant. Using linear transformation of the B regression coefficients, were able to assign points for the risk factors. It becomes apparent that the strongest predictors are Gleason >7 and PSA >20. The points allowed us to determine risk groups for failure based on scores ≤2 (low risk), 2–4 (intermediate risk), and >4 (high risk). Low-risk factors were Gleason <7, PSA <10, and EPE/mar negative. Intermediate-risk factors were Gleason 7, PSA 10–20 ng/ml, and EPE/margin+, and high-risk factors were Gleason >7 and PSA >20 ng/ml. Low risk for recurrence (the low-risk group) was no more than one intermediate-risk factor, intermediate risk for recurrence (the intermediate-risk group) was two intermediate-risk findings, and high risk for recurrence was all three intermediate-risk factors or Gleason >7 or PSA >20 ng/ml. The majority of these surgery patients (64%) were classified as low risk (Table 4.)

**Figure 2:**
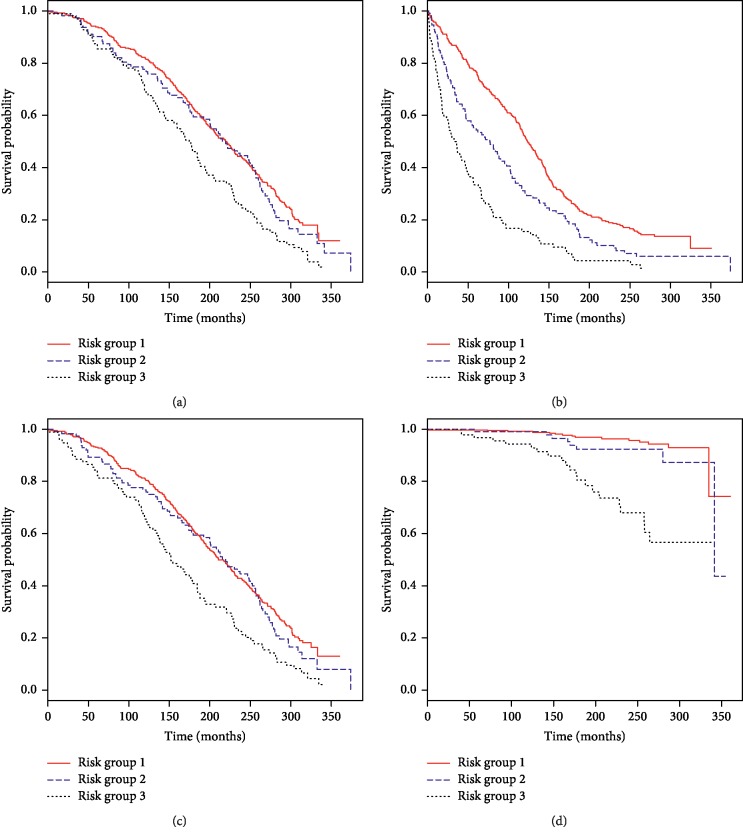
Kaplan–Meier estimates for (a) overall survival, (b) biochemical failure-free survival, (c) metastatic failure-free survival, and (d) cancer-specific survival by risk groups.

Based on the risk groups, 23%, 50%, and 72% of the low, intermediate, and high risk groups, respectively, suffered from biochemical failure. More importantly, 3%, 5%, and 19%, respectively, developed metastatic disease and 4%, 7%, and 22%, respectively, died of prostate cancer (Table 5, Figure 2). For metastatic disease and prostate cancer death, there was no significance between groups 1 and 2.

On Cox proportional hazard modeling, Gleason score, pre PSA value, and EPE/margin positive were all statistically significant for metastatic disease and prostate cancer death (*p* value <0.0001 to 0.007).

## 5. Discussion

This cohort of patients dates back to the start of the PSA era (circa 1988), so it represents the longest biochemical follow-up possible. The median/mean age of our patients at the time of surgery was approximately 67 years. According to life tables from 1990 [5], the expected survival for a 65-year-old man was 14 years (to age 79). The median survival of our patients was 84 years, indicating that, in patients fit enough for a major surgery, there was no excess risk of death from the prostate cancer. Indeed, the overall cancer death rate was quite low at 8%, almost half of which were isolated to high-risk patients. The risk of biochemical failure was pervasive, with the majority of the patients having experienced that event on Kaplan–Meier analysis. In spite of that, the risk of overt metastatic disease was relatively low at 6% and remained steady throughout the follow-up period, with about half occurring before and half occurring after 10 years. As noted previously [1], especially with other serious medical issues developing, cancer-specific follow-up became less frequent. This probably contributed to the flattening of the biochemical failure curve later on (Figure 1).

The risk factors (PSA level, Gleason score, and pathologic stage) for biochemical recurrence are well known [2]. We were able to confirm that not only were these factors predictive of biochemical (PSA) failure but also for metastatic disease and death. A higher PSA, a higher Gleason score, and the finding of unfavorable pathology with extraprostatic extension and/or positive margins predicted for biochemical failure, but only Gleason 8–10 and/or PSA >20 ng/ml were predictive of metastatic disease and cancer death. This should be reassuring for the vast majority of the patients without high-risk findings that even if they have a PSA failure, the chance of prostate cancer death is still low. For the most part, this confirms that PSA is not a surrogate [6] for cancer survival. Still, for the high-risk patients, given the higher death rate, closer monitoring, and consideration of intervention is warranted.

As noted, the risk factors for biochemical recurrence are well documented [2], although there is less information for prostate cancer death. Part of the problem is the long natural history of prostate cancer, and most studies are of too short a duration to adequately measure that endpoint.

There are relatively few studies with truly long-term follow-up after prostatectomy, especially in the PSA era. The largest was a composite of 4 sites with the goal of developing a nomogram, which was validated by data from a fifth site [7]. Fifteen-year prostate cancer-specific mortality was reported, although the 4-site group only had a median follow-up of 56 months (and the comparator site 96 months). There were enough patients with longer follow-up to likely make the 15-year data relevant. In the risk analysis, they confirmed that lymphatic and seminal vesicle involvement were significant risk factors for failure and death. Surprisingly, they did not find that the preoperative PSA was predictive, which is contrary to numerous reports [2]. This is likely because patients with a PSA >8 ng/ml were not included. This also would reflect fairly stringent patient selection that may not be relevant to a general population. Other than seminal vesicle involvement, they reported that Gleason 8–10 was a prime determinant of prostate cancer-specific mortality. Combining all the patients (23,910), they reported the results by age groups (<60, 60–69, and 70–79 years). Looking at the single factors, cancer-specific mortality at 15 years was 4.2–11% for Gleason 7, 26–37% for Gleason 8–10, and 2.9–10% for extraprostatic extension. The 20-year mortality was Gleason 7, 9–23%; Gleason 8–10, 31–39%; and extraprostatic extension 6.6–20%. For the Gleason 6 cancer patients, at both 15 and 20 years, the mortality was 0.6 to 1.2%, respectively. These results were similar to what we saw with longer follow-up, with the low- and intermediate-risk groups having a 4% and 7% cancer-specific mortality, respectively, and the high-risk with a 22% cancer-specific mortality. This is in spite of the fact that 25% of our patients had a PSA >10 ng/ml and were considerably older (median age 67 years compared to 60 years in the above study).

We were able to identify high-risk patients (Gleason 8–10 and/or PSA >20 ng/ml) that were at a significantly higher risk of failure and death. Even with that, “only” 22% died of prostate cancer. This clearly indicates that there are other factors that determine the risk of death other than the standard pathology descriptors.

In addition, there is currently a pervasive argument that patients with low-risk clinical factors (Gleason <7, PSA <10 ng/ml, and minimally palpable disease) do not need treatment at all. We and the authors sited above have shown that even with negative pathologic findings at the time of surgery, some of these “low-risk” patients die of prostate cancer. This has been seen in some of the larger active surveillance studies [8]. The message here is that in these perceived low-risk patients, the risk of prostate cancer death is very low, but not zero.

Of course, our data are limited by the retrospective data and the lack of uniform follow-up in the years further out from the surgery. It is challenging to obtain accurate follow-up in very elderly patients. Often their cancer history is just a footnote. Not inappropriately, they are not having routine PSA testing or scanning. This is especially true for the debilitated nursing home patients where even something as significant as bone metastasis may go unrecognized. It was difficult to ascertain from our cohort, but we could identify a few patients with late symptomatic recurrences that might have been prevented by ongoing cancer surveillance and earlier intervention. While it is possible that there were others that suffered from unrecognized metastatic disease, it is likely to be a small number. In our low-and intermediate-risk patients, the number that developed metastatic disease after 10 years was very low (9/485, 2%). All of them except one had a known rising PSA well before 10 years. We think it is reasonable to follow these lower risk patients with PSA every 6 months for the first 4–5 years, annually out to 10 years and then stop if it is still undetectable. The high-risk patient had higher (8/92, 8%) late (>10 years) metastatic failures. Although not exorbitant, it would be prudent to check the PSA annually or biennially for another 5 years (out to 15). We had only 4 patients with metastatic disease recognized after 15 years, and arguably the PSA would have picked up the recurrence long before then.

Our truly long-term data confirm that the risk of dying of prostate cancer after prostatectomy is relatively low (<10% overall). We were able to identify patients at a significantly higher risk that warrant longer term follow-up. Lower risk patients do not appear to need routine follow-up after 10 years.

## Figures and Tables

**Table 1:** Patients' characteristics.

*N* = 581	Median/mean
Age at surgery (years)	66.7/67.2 (62.5–71.3)

Gleason	*N* (%)
<7	210 (36%)
=7	331 (57%)
>7	40 (7%)

PSA	
<10	433 (75%)
>10–20	107 (18%)
>20	41 (7%)

EPE/margin positive	
None	434 (75%)
At least one	147 (25%)

Epe = extraprostatic extension.

**Table 2:** Univariate analysis for the biochemical-free survival (*n* = 581).

Parameters	Risk of failure	HR (95% CI)	*p* value
PSA			<0.0001
<10	28.47	Reference	
10–20	54.21	1.50 (1.198, 1.883)	
>20	75.61	3.97 (2.856, 5.527)	

Gleason score			<0.0001
<7	27.75	Reference	
=7	37.76	1.41 (1.164, 1.706)	
>7	72.5	2.85 (1.990, 4.086)	

EPE/margin			<0.0001
All negative	28.64	Reference	
At least one positive	59.86	1.64 (0.342, 1.998)	

**Table 3:** Multivariate Cox proportional hazard analysis of the scoring system (*n* = 581).

Risk factors	Hazard ratio (95% CI)	*p* value	Regression coefficient	Points^*∗*^
Gleason score				
<7				0
=7	1.35 (1.115, 1.642)	0.0022	0.30	2
>7	2.43 (1.689, 3.498)	<0.0001	0.89	6

Pre-PSA score				
<10				0
10–20	1.39 (1.110, 1.749)	0.0042	0.33	2
>20	3.92 (2.805, 5.469)	<0.0001	1.37	8

EPE and margin				
None				0
At least one positive	1.50 (1.223, 1.833)	<0.0001	0.40	2

^*∗*^Assignment of points to risk factors was based on a linear transformation of the *β* regression coefficients. The coefficient of each variable was divided by 0.3, multiplied by a constant (1.88), and rounded to the nearest integer. Total point is 20.

**Table 4:** Risk for biochemical (PSA) failure based on risk groups.

Risk groups		# patients (%)
Low	No or one intermediate-risk factor	373 (64%)
Intermediate	Two intermediate-risk factors	112 (19%)
High	Gleason >7 or PSA >20 or all 3 intermediate-risk factors	96 (17%)

Low-risk factors: Gleason <7, PSA <10 ng/ml, and mar/epe negative. Intermediate-risk factors: Gleason 7, PSA 10–20 ng/ml, and mar/epe positive. High-risk factors: Gleason 8–10 and PSA >20 ng/ml.

**Table 5:** Biochemical (PSA) failure, metastatic disease, prostate cancer death, and overall survival by risk groups.

Risk group (number)	Biochemical failure (%)	Metastatic disease (%)	Died of prostate cancer (%)	Died (%)
Low (373)	87 (23%)	12 (3%)	15 (4%)	280 (75%)
IM (112)	56 (50%)	6 (5%)	8 (7%)	91 (81%)
High (96)	69 (72%)	18 (19%)	21 (22%)	87 (91%)
Total (581)	212 (36%)	36 (6%)	44 (8%)	458 (79%)

IM = intermediate.

## Data Availability

The data are stored in a secure database in our institution.

## Supplementary Materials

Supplementary Materials Methodology for long-term follow-up.
