# Supplementary material for: Long-Term Follow-Up after Prostatectomy for Prostate Cancer and the Need for Active Monitoring
Source: Prostate Cancer. 2020 Mar 10;2020:7196189. doi: 10.1155/2020/7196189 (PMC7085821; doi:10.1155/2020/7196189)
Supplement: Supplementary Materials — Methodology for long-term follow-up. [file 7196189.f1.docx]

SUPPLEMENT

Follow up details

The ideal situation for determining the outcome of any disease is when patients are seen on a uniform regular basis, with systematic determination of disease status. The most universally accepted measure in prostate cancer is that of PSA. Historically, the routine obtaining of cancer specific imaging (bone scans and CT scans) in follow up has been abandoned in favor of symptom and PSA driven indicators. As a regional referral center, the patients seen were a mixture of local residents and those from numerous outside facilities at varying distances. With six different urologists, there was no uniform regimen for follow up, but typically, after the first post-operative year, patients would be followed every 6 months for 4-5 years and if doing well, released back to their primary physician after that. For local patients, this was usually accomplished unless the patient missed an appointment and did not reschedule. Often this was from an interceding medical problem, which usurped an increasingly remote cancer history. For outside patients follow up in house varied from a single visit to semi-regular visits, although usually less than semiannually. Correspondence regarding those visits was submitted to the outside physicians. For patients who continued their follow up care with their local physician, no effort was made to dictate how that was done. Those physicians rarely sent follow up information back. Some patients would return intermittently over the years, usually for a different problem, but rarely was their cancer follow up history pursued nor were PSAs obtained on that visit by the non-urologists or oncologists that saw them.

The data base was initiated in 1994 by one of the co-authors (GPS) and maintained (updated) regularly through the 1990’s. Those core medical records no longer exist. In the early 2000’s an electronic data base was obtained (EMRx*), but with the acquisition of EPIC^†^ (2014), that database was closed and is no longer accessible. Some data was loaded as “historical data” in EPIC, but no notes prior to 2002 and no laboratory data prior to 2004 has been maintained. In 2004-2006, two of the coauthors (GPS and MH) updated with the database from the existing electronic medical records and with attempts at getting records from the outside physicians. For patients not seen in the previous two years, correspondence was sent to the last known outside physician. There were mostly the family physicians. If none could be identified, a letter was sent to the patient requesting follow up, with the name of their current physician and permission to contact them. The response in all cases was variable. For non-response one of the co-authors (GPS) would call them. Between the phone calls and the letters, the minimum requests were: is the patient alive and well?, did they ever have a recurrence of prostate cancer?, any known hormone or radiation treatment?, any and all PSAs?, if dead, did they die of prostate cancer, any other physician that might have this information? Not surprisingly, the information was spotty, but we trusted death dates (for overall survival) and felt fairly sure we could document who died of prostate cancer (for cancer specific survival). If PSAs had been obtained in the previous 5 years, we were fairly confident we obtained at least some because we tried hard to determine who might have done them. It was interesting, but probably not surprising or inappropriate that now more than 10 years out, PSA levels were rarely routinely obtained. Imaging was driven based on symptoms and the obtaining of PSA frequently accompanied that indication. We were confident that their primary physicians would be aware if patients were on androgen ablation or had received radiation, but the details were often lacking. Going back to try to find the source information was difficult, especially if the patient died (records are not routinely retained in the private offices). We think we fairly well captured failure (based on treatment). The initial manuscript was published based on this data (1).

For the current analysis (2016-2019), our goal was to obtain follow up until death, or if still alive, for the most recent 2 years (through 2017).

First, we screened our existing data set and updated it from our current medical records as well as the local Veterans Association (VA) facility. We collected all available data (including PSA if done).

For the remaining patients, the first goal was to determine whether the patient was still alive. Resources used:

1. Internet search. Primarily by patient name, including on-line white pages search, online obituaries, etc.
2. Social security death index search
3. Contact with last known outside provider

If discovered deceased or uncertain:

1. Attempted to get information from last known physician, failing that:
2. Via death certificates (from the stat of last known residence)

Although we identified that we were physicians trying to get follow up for our own patient (which would be continuity of care for HIPPA purposes), the willingness of the different states to provide information was variable.

- Texas- were able to register as a researcher and have the database searched at significant cost. Search for 244 individuals requested, some known to be deceased and some unknown. Interesting that in some in whom we knew from a found obituary were deceased in state, they did not always have a filed the death certificate. There were 10 known deceased patients without a death certificate. It is possible they had moved our of state. Overall, we obtained 159 death certificates.
- Florida- we registered as researchers to get the information on 2 patients (cost)
- Arizona- connected us with a senior researcher who graciously provided us with the information we needed on two patients (without sending certificates- no cost)
- Indiana (2), Missouri (1), and Wisconsin (2) will willing to send a death certificate (at cost)
- California and Oklahoma refused without a signed release (from dead patients).

In the end, of those still alive (n= 122 ), 88 (72%) had active follow up in the last two years and 109 (89%) had active follow up in the last 5 years. Overall, for all patients, 69% had a PSA within the last 5 years and 89% within the last 10 years of their most recent follow up (dead or alive).

* Synthesys Technologies Inc,Austin, Texas USA

† Epic, Verona, Wisconsin, USA
